# Microevolution of Pandemic *Vibrio parahaemolyticus* Assessed by the Number of Repeat Units in Short Sequence Tandem Repeat Regions

**DOI:** 10.1371/journal.pone.0030823

**Published:** 2012-01-24

**Authors:** Katherine García, Ronnie G. Gavilán, Manfred G. Höfle, Jaime Martínez-Urtaza, Romilio T. Espejo

**Affiliations:** 1 Instituto de Nutrición y Tecnología de los Alimentos (INTA), Universidad de Chile, Santiago, Chile; 2 Instituto de Acuicultura, Universidad de Santiago de Compostela, Campus Universitario Sur, Santiago de Compostela, Spain; 3 Department of Vaccinology and Applied Microbiology, Helmholtz Centre for Infection Research (HZI), Braunschweig, Germany; Naval Research Laboratory, United States of America

## Abstract

The emergence of the pandemic strain *Vibrio parahaemolyticus* O3:K6 in 1996 caused a large increase of diarrhea outbreaks related to seafood consumption in Southeast Asia, and later worldwide. Isolates of this strain constitutes a clonal complex, and their effectual differentiation is possible by comparison of their variable number tandem repeats (VNTRs). The differentiation of the isolates by the differences in VNTRs will allow inferring the population dynamics and microevolution of this strain but this requires knowing the rate and mechanism of VNTRs' variation. Our study of mutants obtained after serial cultivation of clones showed that mutation rates of the six VNTRs examined are on the order of 10^−4^ mutant per generation and that difference increases by stepwise addition of single mutations. The single stepwise mutation (SSM) was deduced because mutants with 1, 2, 3, or more repeat unit deletions or insertions follow a geometric distribution. Plausible phylogenetic trees are obtained when, according to SSM, the genetic distance between clusters with different number of repeats is assessed by the absolute differences in repeats. Using this approach, mutants originated from different isolates of pandemic *V. parahaemolyticus* after serial cultivation are clustered with their parental isolates. Additionally, isolates of pandemic *V. parahaemolyticus* from Southeast Asia, Tokyo, and northern and southern Chile are clustered according their geographical origin. The deepest split in these four populations is observed between the Tokyo and southern Chile populations. We conclude that proper phylogenetic relations and successful tracing of pandemic *V. parahaemolyticus* requires measuring the differences between isolates by the absolute number of repeats in the VNTRs considered.

## Introduction

Diarrhea associated with seafood consumption is caused primarily by pathogenic *Vibrio parahaemolyticus*. This species includes a large number of marine strains, only a few of which are pathogenic in humans [1]. Cases of diarrhea related to seafood consumption increased worldwide with the emergence of pandemic strain O3:K6, which was originally observed in Southeast Asia [2]. Isolates in this group are commonly recognized by genetic markers, including the O3:K6 antigens, which determine the serovar; the presence of genes *toxRS/new* [3], *orf8* [4], and *tdh*; and the absence of *trh*, which is found in some pathogenic strains. Isolates with this genotype correspond to a clonal complex, whose independently obtained isolates are rarely differentiated. The identity of the genomes is assessed by multilocus sequence typing (MLST) [5], [6], genome restriction fragment length polymorphism–pulsed field gel electrophoresis (RFLP-PFGE) [7], direct genome restriction enzyme analysis (DGREA) [8], and arbitrarily primed polymerase chain reaction (AP-PCR) [2], [3]. Some variants have been observed occasionally, namely in serotype [9] but also in RFLP-PFGE or AP-PCR patterns [2], [10], and others lacking *toxRS/new* or *orf8* [10]. Microarray-based comparative genomic hybridization (M-GCH) of 4021 genes allowed the differentiation of 39 pandemic strains into five subgroups [11]. The M-GCH data (log2 ratios) for these five subgroups vary in the genomic islands and O/K antigen genes. Despite the resolving power of these last methods, however, the discrimination is not sufficient for tracing the isolate in seafood outbreaks caused by pandemic *V. parahaemolyticus*. Multiple locus variable analysis (MLVA) of the variable number tandem repeats (VNTRs) has been developed for most medically relevant bacterial species and can be used effectively for tracing outbreaks or other forms of bacterial dissemination [12], [13]. VNTRs consist of short sequences, known as repeat units or motifs, that are repeated in tandem and have been shown to vary in repeat copy number by the insertion or deletion of one or more repeat units. Although recombination that produces large differences also occurs, it is less frequent [14]. This system has been highly successful for epidemiological studies of genetically homogeneous bacterial pathogens, such as *Yersinia pestis* [15], *V. cholerae* O1 and O139 [16], *Escherichia coli* O157:H7 [17], *Bartonella henselae* [18], and *Mycobacterium leprae* [19], providing useful genetic discrimination whether the populations were worldwide, regional, or from a local outbreak. MLVA of VNTRs has also been employed to study within-host evolution of *Burkholderia pseudomallei* infection [20]. The high-resolution power of MLVA was recently shown for pandemic *V. parahaemolyticus*; eight VNTRs in 28 pandemic *V. parahaemolyticus* O3:K6 strains isolated from human cases produced 28 distinct VNTR patterns [21]. Analysis of 36 pandemic isolates belonging to the clonal complex isolated in Chile produced 26 patterns [22]. Multiple *loci* VNTR analysis of a number of representative pandemic *V*. *parahaemolyticus* strains from Asia, Peru, and Chile using seven polymorphic *loci* divided the populations into two genetically distinct groups [23]. One of them grouped with the original Asiatic population and strains arriving in Peru and Chile in 1997 comprised one group. Thus, MLVA seems useful for tracing and studying the phylogeny within highly homogenous bacterial species subgroups. However, a more exact interpretation of the results requires knowing the rate and mechanism of variation of the VNTRs. Here, we show that the variation of VNTRs is better explained by single stepwise mutation (SSM) and that a more credible phylogeny by comparing VNTRs is obtained when data is analyzed considering that variation between VNTRs occurs by SSM. We also show that the analysis of the relation between variants generated by SSM show plausible results using the Minimum Spanning Trees (MST) method with the Manhattan category which builds the tree considering the sum of the differences in repeat units of any two mutants [24]. MST is a mathematical topology tool that applies the maximum parsimony principle, applicable for population modeling (micro-evolution) and epidemiology. When a set of distances is given between n samples, a minimum spanning tree is the tree that connects all samples in such a way that the summed distance of all branches of the tree is minimized.

## Materials and Methods

### Clones and Native Bacterial Strains

Four different clones of pandemic *V. parahaemolyticus* were used for serial cultivation and subsequent MLVA analysis. Clones are designated those cultures obtained from single colonies of strains VpKX and PMC57.5 used for *in vitro* study of the mutation rates. Native strains were isolates with pandemic *V. parahaemolyticus* characteristics, and were obtained from clinical and environmental samples from different geographical sites. Clone KX-1 was obtained from a sample of the reference prototype strain RIMD2210633 (VpKX), isolated in 1996 and sequenced [25]. It was received in 2005 from Professor Takeshi Honda (Research Institute for Microbial Diseases, Osaka University) and kept in soft agar from this year [4]. Clones PMC57.5-1, -2, and -3 were obtained from PMC57.5, isolated in 2005 from a clinical sample in Puerto Montt, Region de Los Lagos, and kept in soft agar at room temperature. The pandemic strains (between 1995 and 1998) from Southeast Asia used in this study were received from Mitsuaki Nishibuchi (Center for Southeast Asian Studies, Kyoto University). Chilean native strains were collected from the coast of Antofagasta (1998) in the north and from Region de Los Lagos in the south (between 2004 and 2009). Most of the isolates were described in previous publications [8], [26], [27], [28], [29]. Data for the pandemic strains from Tokyo correspond to those reported by Kimura *et al* [21]. These strains are described in Table S1.

### Serial Subcultures

Serial subculturing was performed as described previously [30]. Briefly, clones KX-1) and PMC57.5-1 were suspended in 5 mL of LB with 3% NaCl and incubated at 37°C in a rotary shaker overnight. Each culture was serially propagated into 20 subcultures diluted 1∶100 plus 80 subcultures diluted 1∶10000. After 100 subcultures, performed in 100 days, 60 colonies from each culture were picked up for MLVA.

### Parallel Serial Passage Experiments (PSPE)

PSPE were performed as described previously [15]. Two single colonies from strain PMC57.5 obtained in LB agar 3% NaCl (clones PMC57.5-2 and PMC57.5-3) were grown independently in 5 mL of LB with 3% NaCl, and a single colony from each of these cultures was serially transferred for 10 passages. In both types of *in vitro* mutants, from subcultures and from PSPE, the number of total generations was calculated as follows: number of colonies x number of duplications for each colony per subculture x number of subcultures.

### DNA Extraction and MLVA of bacterial clones, *in vitro* mutants and native bacterial strains

DNA was extracted with the DNA Wizard Genomic kit (Promega, Madison,WI). PCR reactions and analysis of the amplicon size were performed as described previously [22] except that only six VNTR loci (VNTRs 1, 3, 5, 6, 7 and 8) were selected for determination of mutation rates (Table 1) and each VNTR was amplified independently. When 8 VNTR loci (VNTRs 1 to 8) were amplified for the phylogenetic study of the native strains three multiplex reactions were performed. MultiA (VNTR 2 and 7), MultiB (VNTR 1 and 8) and MultiC (VNTR 2, 3, 4, and 6).

**Table 1:** Number of repeat units of six VNTR loci in four clones obtained from pandemic *Vibrio parahaemolyticus* strains VpKX and PMC57.5 and mutation rates after prolonged in vitro culturing.

		Serial cultures				Parallel serial passages			
VNTR	Repeat Motif	KX-1		PMC57.5-1		PMC57.5-2		PMC57.5-3	
		Repeat units	Mutat rate	Repeat units	Mutat rate	Repeat units	Mutat rate	Repeat units	Mutat rate
**1**	ATAGAG	28	3.7	34	4.2	38	6.0	6	0.0
**3**	ATCTGT	7	0.0	7	0.8	7	0.0	7	0.0
**5**	CTCAAA	7	0.0	7	0.4	7	0.0	7	2.4
**6**	GCTCTG	17	0.1	14	0.7	14	3.8	18	2.4
**7**	CTGCTC	6	0.0	6	0.3	6	0.0	6	0.8
**8**	CTTCTG	7	0.0	7	0.4	7	0.0	5	0.0
**Combined Mutation rate**			**3.8**		**6.7**		**8.8**		**5.6**

Mutat rate: Mutation rate (x 10^4^).

### Mutation Rate and Analysis of Mutation Type

Mutation rate was calculated as: number of mutants/number of generations. The distribution of VNTR mutants with 1, 2, 3, or more repeat units deleted or inserted was calculated from the combined data for the populations from the four clones of pandemic *V. parahaemolyticus* examined. A geometric distribution was obtained with the experimental data of the number of mutants that differed in one repeat unit from the closest neighbor that was coincident with the theoretical expected distribution, according to the equation P(X = n)  =  P(1-P)^n-1^ [31], where P is the probability of a single repeat mutation, and n is number of repeats involved in a mutation. Only mutants that differed in less than three repeat units from their closer neighbors were considered. Mutations that occur simultaneously in more than one repeat unit were assumed to be produced by recombination [32].

### Genetic Distance

Genetic distance, (δµ)^2^, of allele size in populations computed as δµ^2^  =  (µ_A_–µ_B_)^2^ where µ_A_ y µ_B_ are the variances and means, respectively, was defined previously for microsatellite loci, incorporating the features of stepwise mutation model [33] by including the difference in each VNTR, by using Microsatellite Analyzer (MSA) version 4.05 software. The neighbor-joining tree-building method of MEGA4 [34] software was used to infer the phylogenetic tree based on genetic distance.

### Phylogenetic Relationship analysis of *in vitro* Mutants or Native Variants

The minimum spanning tree (MST) was calculated for the different populations of the mutants obtained *in vitro*, and from the native bacterial strains, by using the Manhattan coefficient (offset = 0, saturation = 3) in Bionumerics (v 5.10, Applied Maths, Sint-Martens-Latern, Belgium) [24].

## Results

### Diversity of VNTRs among Clones of the Same Strain and Mutation Rates

**Figure 1:**
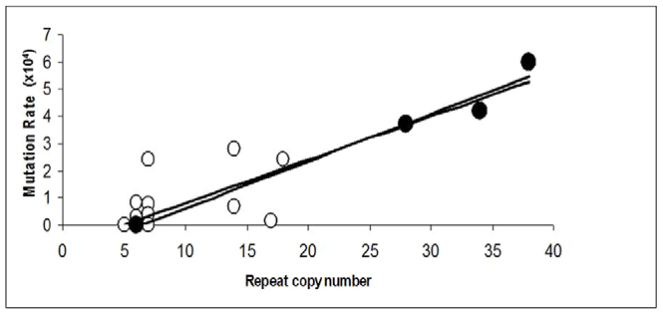
Mutation rates of VNTRs of pandemic *V parahaemolyticus,* in relation to the number of repeats in each VNTR. White circles correspond to the mutation rates observed for any VNTR, circles in black correspond to the mutation rates observed for VNTR1 in the different clones. The best fit equation for VNTR1 was y = 0.1736×–1.1245 (R^2^ = 0.9674) while that for all the VNTRs was y =  0.1598×– 0.7923 (R^2^ = 0.8052).

We measured the mutation rates of six VNTRs for four clones, obtained by culturing single colonies of *V. parahaemolyticus* pandemic strains. One of these clones, KX-1, was obtained from a sample of strain RIMD2210633 (VpKX), which was received in 2005 and has been sequenced [25]. This clone (KX-1) showed the same repeat numbers reported for the published genome sequence in each analyzed VNTR, except in VNTR3, which contained seven repeat units instead of the six reported in the genome sequence. The other three clones were derived from strain PMC57.5, which was obtained from a clinical sample in southern Chile in 2005 and purified twice by selecting a single colony after plating [8]. Although strain PMC57.5 is indistinguishable from VpKX by the common genetic markers of the pandemic strain, as well as by RFLP-PFGE and DGREA patterns [8], examined clones from this strain differed from VpKX and among themselves in several VNTRs. Ten clones derived from PMC57.5 were randomly selected to explore their possible variation in VNTRs; among them, eight contained 34 and 14 repeat units in VNTRs 1 and 6, respectively; one (PMC57.5-2) contained 38 repeat units in VNTR1; and another (PMC57.5-3) contained six repeat units. This last clone also differed from the other clones of the same strain by the presence of 18 and 5 repeat units in VNTRs 6 and 8, respectively, instead of the 14 and 7 repeat units found in the other clones (Table 1). Because VpKX and the PMC57.5 strains had been kept in sealed stab agar at room temperature since 2005, the observed diversity among the clones derived from the same sample may be due to growth during storage; in some *Salmonella spp.*, such phenomenon has been observed [35], [36]. Mutation rates were measured in experiments performed by either serial subculturing [2] or a series of PSPEs [15]. Mutation rates differed between both VNTRs and clones, which was apparently related to the number of repeats in the VNTR in each clone (Figure 1). The relationship between the number of repeats and mutation rate was described previously [31], [32], but this is not precise when different VNTR are considered. The relationship became more precise when the same VNTR with different numbers of repeat units is considered (note the values of R presented in the legend of Figure 1 for each situation). The low number of repeat units in VNTR1 of clone PMC57.5-3 is the probable cause of the much smaller mutation rate observed for VNTR1 in this clone.

### Diversity in the Population Obtained after Serial Cultivation

**Figure 2:**
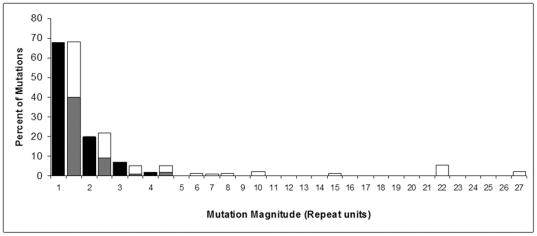
Frequency of mutants with increasing differences in the number of repeat units. Black bars correspond to percent of predicted mutants. Combined bars correspond to percentage of observed mutants, white sections of bars correspond to deletions and gray to insertions.

The distribution of mutation types observed for the different pandemic *V. parahaemolyticus* clones was consistent with the distributions observed in *E. coli* O157:H7 [17] and *Y. pestis* [32]. The frequency of mutants with 1, 2, 3, or 4 repeat unit changes roughly followed a geometric distribution. Though deviations were observed in some experiments, the mean distribution of the four clones showed fairly good agreement with geometric distribution (Table 2, Figure 2). As observed in previous studies [2], [30], we found both an unexpected high proportion of insertions and a small number of mutants with large changes in repeat units in the VNTRs (Figure 2). As previously postulated for clonal populations including similar generation numbers, mutants with changes smaller than four repeat units may be generated by SSM in which mutations occur by the insertion or deletion of single units, as initially proposed for microsatellites in eukaryotes [37] and later for bacteria [32]. On the other hand, mutants with large changes in repeat units, unlikely to be observed if the distribution were ideally geometric are probably generated by recombination. Overall, our results support the theory advanced for *E. coli* O157:H7 [31] and *Y. pestis* [32], that mutations in VNTRs mostly occur by SSM.

**Table 2:** Frequency of observed and expected mutants according to a geometric distribution, with 1, 2, 3, or 4 repeat unit changes for each of the clones examined.

Repeat unit changes	KX-1	PMC57.5-1	PMC57.5-2	PMC57.5-3	Mean
1	64 ***-***	74 ***-***	86 ***-***	47 ***-***	68 ***-***
2	25 ***23***	23 ***19***	7 ***12***	33 ***25***	22 ***20***
3	11 ***8***	3 ***5***	0 ***2***	7 ***13***	5 ***7***
4	0 ***0***	0 ***0***	7 ***0***	13 ***7***	5 ***2***

The expected frequency of mutants according to geometric distribution is shown in bold italics.

### Evolutionary Models and Phylogenetic Trees of in vitro mutants

**Figure 3:**
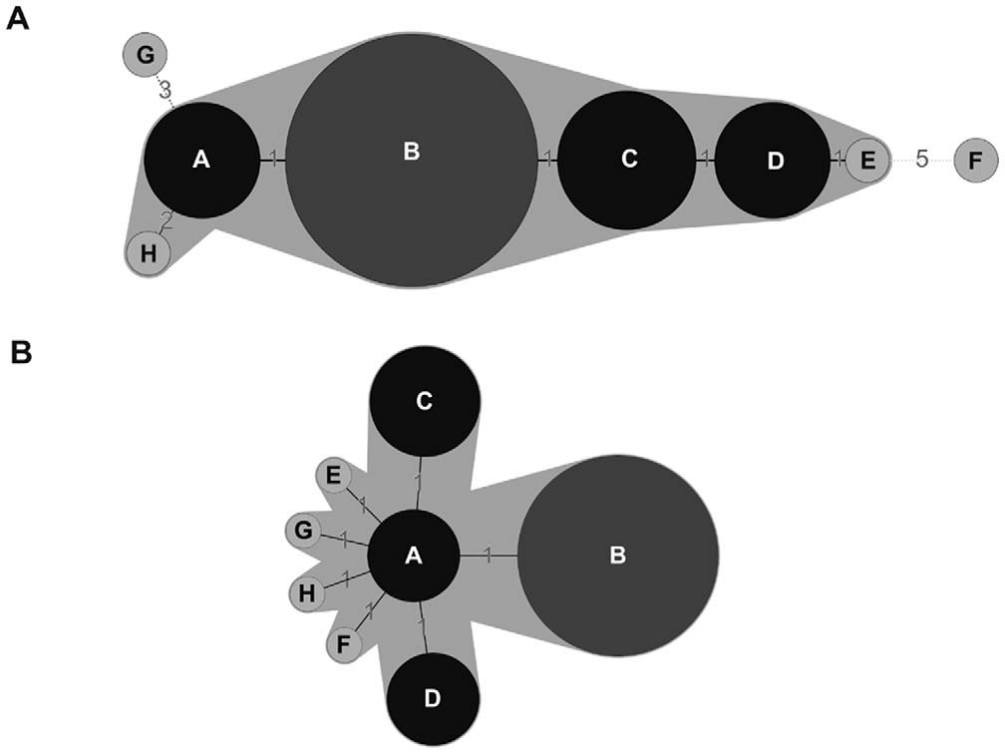
Phylogenetic trees for KX-1 population obtained by MST-Manhattan (A) and MST categorical (B). Each circle corresponds to the different cluster of mutants. Numbers in the lines correspond to the differences in VNTRs repeat units between clusters.

**Figure 4:**
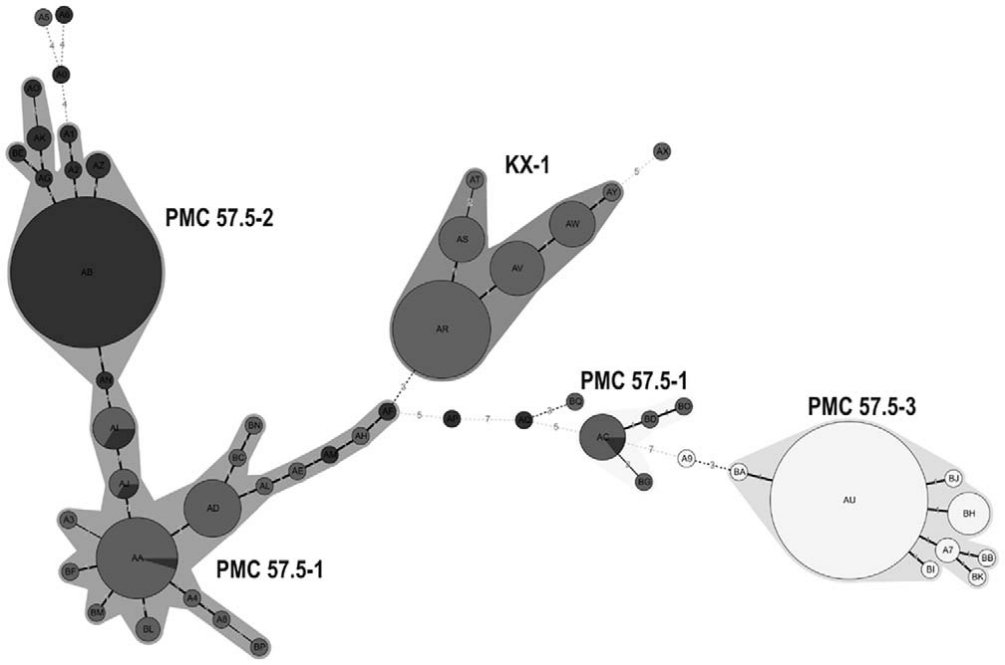
Phylogenetic tree of the mutants from the populations of the four different clones obtained after serial subculturing of pandemic *V. parahaemolyticus*. Tree was obtained by MST-Manhattan. The founder of each cluster is indicated above the clusters.

Taking into consideration that most mutants emerge by SSM, we constructed phylogenetic trees of the mutants observed after prolonged cultivation by considering the absolute differences in the number of repeats in the different VNTRs. For this reason we employed Minimum Spanning Tree (MST) using the Manhattan category which builds the tree considering the sum of the differences in repeat units of any two mutants. If the absolute value of repeat units is not considered, using by example a categorical approach in MST, or the categorical approach of multilocus sequence typing (MLST), mutant pairs with different number of repeat units appear equally distant. The difference in the phylogenetic trees when these different coefficients are employed is evident in Figure 3 which shows the MST tree obtained for the KX-1 population of 75,600 generations using either the Manhattan (Figure 3A) or the categorical approach (Figure 3B). Cluster B correspond to the founder. However, when the absolute difference in the number of repeat units is not considered, the founder is not properly identified and distantly related mutants are shown as closely related or considered direct variants (Figure 3B). In contrast, the tree obtained by considering the absolute difference in repeat units shows only mutants differing in a single repeat unit as direct variants, as would be expected if mutants are generated by SSM. Because one potential application of the MLVA of *V. parahaemolyticus* is the tracing of outbreaks, we analyzed the combined four populations obtained from each the four original clones after serial passages in the laboratory using the Manhattan approach. Despite the small differences in VNTRs among the founders, we correlated almost every mutant with its actual founder, distinguishing between populations with different but closely related founders (Figure 4). If the analysis is performed with the categorical approach, only a few mutants derived from different founders were distinguished (data not shown).

### Phylogeny of Native Populations

**Figure 5:**
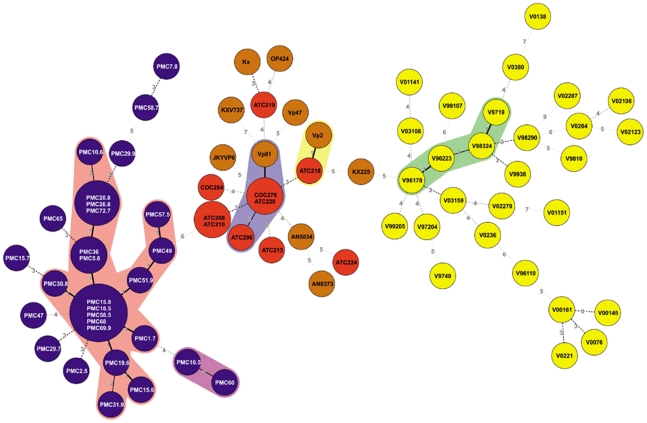
Phylogenetic tree of native isolates of pandemic *V. parahaemolyticus* obtained worldwide. Phylogenetic tree generated by MST-Manhattan for native isolates obtained from southern Chile (blue) northern Chile (red), Southeast Asia (brown) and Tokyo (yellow).

Another useful application of MLVA is population modeling (microevolution) and epidemiology [23], [38]. We used the MST approach to study the relationships between a large number of native strains of *V. parahaemolyti*cus. Eight VNTR loci were used for this analysis (Table S4). Because pandemic clinical strains might represent a subpopulation of the pandemic strain population found in shellfish, we compared 29 clinical strains and 21 environmental strains collected in southern Chile between 2004 and 2009. The analysis did not differentiate between populations of clinical and environmental strains. No differences were observed between strains isolated in different years. We also compared the strains collected in southern Chile; northern Chile, where an outbreak caused by the pandemic strain was observed in 1997 [28]; and Southeast Asia to determine if MLVA (Manhattan approach) allows us to distinguish between strains according to the geographic origin and to establish possible relationships among these populations. We also included the data from a collection of 28 strains isolated in Tokyo and analyzed for the same 8 VNTRs by Kimura *et al* [21]. Using this approach, almost every mutant from these four populations were clustered according to their geographic origin (Figure 5). In accordance with previous results [23], populations from the outbreak in northern Chile seem to be more closely related to the Southeast Asian population than the population from southern Chile. Interestingly, the Japanese population consisting of mostly clinical strains isolated from single patients in Tokyo between 1996 and 2003 [21] clustered separately from isolates obtained in different Southeast Asia locations.

**Figure 6:**
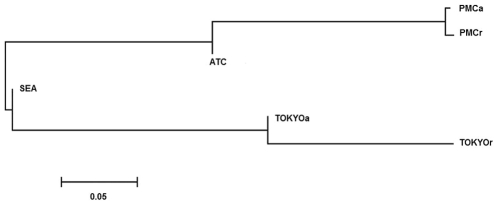
Neighbor Joining tree for different populations of pandemic *V. parahaemolyticus*. Neighbor joining tree based on (δµ)^2^ genetic distance for population of clinical isolates from southern Chile (PMCa isolated from 2004–2006 and PMCr isolated from 2007–2009), northern Chile (ATC isolated from 1997–1998), Southeast Asia (isolated from 1996–1998) and Tokyo (TOKYOa isolated 1996–1999 and TOKYOr from 2000–2003).

Genetic distance (δµ)^2^ has been used to date divergence between mammal populations [39]. We used this parameter that corresponds to the differences between the mean lengths of the VNTRs of each population in order to date the possible split between the four native populations analyzed by MST. Because strains from Tokyo and southern Chile were isolated during a period of 8 and 6 years, respectively, we divided these populations into two (ancient and recent isolates, obtained in the first and second half of the period, respectively). The calculated genetic distances (Table S2) allowed us to construct a tree by neighbor-joining, showing the apparent split of the populations of pandemic *V. parahaemolyticus* from different geographic regions (Figure 6).

## Discussion

The analysis of VNTRs with large mutation rates offers an opportunity to study evolution in a clonal population, such as pandemic *V. parahaemolyticus*. However, interpretation of the results for microevolution and population epidemiology requires knowledge of the mutation rates and understanding how VNTRs change with time. The mutation rates we measured in pandemic *V. parahaemolyticus* were similar to those observed in *E. coli* O157:H7 and *Y. pestis* by PSPE; values observed were from 3.4×10^−6^ to 4.0×10^−4^ for *E. coli* and 8.5×10^−6^ to 3.7×10^−4^ for *Y. pestis* [31], [32]. The differences in the number of repeat units between the clone of RIMD2210633 (VpKX) we analyzed, and that from other clones whose genome was sequenced [25] are probably due to subsequent propagation of the strain. More illustrative of the changes in VNTRs is the finding of different clones from purified colonies of *V. parahaemolyticus* stored in agar stabs. The emergence of the variants probably occurred during growth, which is known to occur in bacteria stored in stabs [35], [36]. These observations indicate the need to store strains at a low temperature to avoid growth and to consider the possible consequences of strains stored in stab gels in MLVA results. This consideration should also be taken into account when sequencing the whole genome of stored strains, and in the interpretation of differences between sequenced bacterial genomes.

In this study we determined the relationship between mutation rates and the number of repeats in VNTRs, which was already described in *E. coli* O157:H7 and *Y. pestis* [31], [32], but in this case we are able to demonstrate that this relationship is more evident and precise when the mutation rates observed of the same VNTR from clones with different numbers of repeats are compared. However, it cannot be discarded that the relationship is more precise because the high number of repeat units in the VNTR compared (VNTR1). The mechanism of VNTR variation has been more deeply explored and discussed regarding the variation of tandem repeats in microsatellites in eukaryotes. The accepted mutational mechanism leading to changes in microsatellite length is polymerase template slippage [40], [41]. During the replication of a repetitive region, DNA strands may dissociate and then re-associate incorrectly. Renewed replication in this misaligned state leads to the insertion or deletion of repeat units, altering allele length. The oldest model, and probably the simplest, is SSM, in which the number of repeat units is equally likely to increase or decrease by one at a rate independent of the microsatellite length [37]. Since then a number of models have been proposed to account for the effect of increasing microsatellite length, length limitations, an increase or decrease of more than one repeat in a single mutation event, point mutations that interrupt the repeat chain, and others. One of these models considers SSM and proportional slippage and point mutations [39]. One of the strongest arguments for the SSM model is that the distribution of mutants with 1, 2, or 3 repeat unit differences follows a geometric distribution. Similar to Vogler *et al* [31], [32], we also found a geometric distribution for mutants with 1, 2, or 3 repeat unit differences. Using a different approach, we previously validated the SSM model for a large set of native strains of pandemic *V. parahaemolyticus* by showing that at least 71% of the allelic changes between closest relatives were related to differences in one repeat [23]. Despite the general acceptability of this model, some MLVAs consider differences in each allele (VNTR) in a binary or categorical system without pondering the differences in the number of repeat units. This consideration is only later integrated for deeper analysis of the results, though it has been extensively used for analyzing microsatellite variation [39], [42]. According to a SSM model of VNTR variation, an appropriate method for analyzing MLVA data is the MST of Bionumerics using the Manhattan category; in this way the absolute value of the difference in the number of repeat units is incorporated for construction of the more parsimonious tree. Because Bionumerics is not freely available, goeBURST can be employed with similar results to MST using Manhattan category if each informative repeat unit, instead of each VNTR, is introduced as a locus or allele. goeBURST [43] (http://goeburst.phyloviz.net/) is a modified version of eBURST, a parsimony-based method commonly used to determine the genetic relatedness of bacterial populations that have diverged over short evolutionary time spans using either MLVA or MLST. goeBURST will consider absolute difference of repeat units if each unit is a locus or allele instead of the VNTR, as is usually done. Table S3 contains the data from the KX-1 experiment displayed as should be uploaded in goeBURST. The alternative tree obtained when the absolute difference in repeat units is not considered (categorical, Figure 3B) seems not reliable at first because it shows a similar relationship for pairs differing in one of the VNTRs independent of the difference in the number of repeat units between these pairs. A more objective comparison of the plausibility of both trees was obtained by calculating the overall probability for these alternative trees. When calculated according to Vogler *et al* [32] as P  =  П^n^_i = 1_µ_i_, where µ_i_ is the probability of a given mutation and n is the number of mutational steps, the results were 3.2×10^−35^ for the categorical MST tree versus 8.8×10^−31^ for the Manhattan MST tree, assuming SSM.

Previous Manhattan-MST analysis of 69 strains from Asia, Peru, and Chile distinguished two groups. One group included all strains from Asia and some from Peru, and northern Chile. The second group was composed of strains from Peru and southern Chile [23]. Using a different set of VNTRs and a larger collection of 98 strains, that comprise the strains from Tokyo analyzed by Kimura *et al* [21], we broaden the population epidemiology of pandemic *V. parahaemolyticus* in the present study. The MST-Manhattan showed, as in the previous publication [23], that strains from northern Chile cluster together with Southeast Asia strains and that this cluster is clearly differentiated from strains isolated in southern Chile. Unexpectedly, we found that the strains from Tokyo clearly differentiate from the strains of Southeast Asia. However, the MST tree is probably better interpreted together with the Neighbor -Joining tree based on genetic distance. This parameter, defined as (äµ)^2^, and incorporates features of the SSM, has been used to infer population structure and demographic history in mammals, including humans, according to microsatellite variation [33], [42]. The MST-Manhattan and (δµ)^2^ trees suggest that the strains that caused the outbreaks in northern Chile are closely related to Southeast Asia strains and, thus, probably arrived in northern Chile from Asia. The closeness of the strains found in southern and northern Chile suggests that strains in the south were probably derived from northern strains and do not correspond to an independent introduction of Southeast Asia strains. On the other hand, strains from Tokyo are more closely related to Southeast Asia strains. When interpreting the trees it should be considered that beside geographical origin the strains differ by date of isolation. Differences in the date of isolation seemed to be more clearly observed in strains from Tokyo isolated from 1996 to 1999 (TOKYOa) and from 2000 to 2003 (TOKYOr) (Figure 6). The deepest split in the pandemic populations we analyzed seems to have occurred between the southern Chile and Tokyo populations. Goldstein *et al* [33], [42] used (δµ)^2^ to estimate divergence times in primates according to a relationship between the average mutation rate of microsatellites and the generation time, according to the equation: Eg [ (δµ)^2^ (T)]  = 2β. Assuming that the isolates found in southern Chile between 2004 and 2009 descend from a population that split from the Southeast Asia population isolated between 1996 and 1998 and hence evolved independently for about 10 years, pandemic *V. parahaemolyticus* can be estimated to have been reproducing with a generation time of 3.6 days. Assuming that the genetic distances underlying the tree have a linear variation with time, the split of the Tokyo and southern Chile populations from the Southeast Asia population occurred around the same time. Similarly, the southern Chile population would have split from the northern Chile population approximately 5 years ago.

## Supporting Information

Table S1 Native strains and clones (*) of pandemic ***Vibrio parahaemolyticus*** **used in this work.**
(XLS)

Table S2 **Genetic distance (δµ)^2^ between populations of pandemic** ***V. parahaemolyticus*** **isolated in different years and geographical location.**
(XLS)

Table S3 **Number of repeats in the different VNTRs of the KX-1 population, ready for uploading in goeBURST.**
(XLS)

Table S4 **VNTR profiles of native strains used in this work.**
(XLS)
